# Research trends in peritoneal dialysis literature: a bibliometric and topic-modeling analysis of PubMed publications from 2016 to 2025

**DOI:** 10.12701/jyms.2026.43.58

**Published:** 2026-08-21

**Authors:** Seok Hui Kang

**Affiliations:** Division of Nephrology, Department of Internal Medicine, Yeungnam University College of Medicine, Daegu, Korea

**Keywords:** Bibliometrics, Data mining, Medical informatics, Peritoneal dialysis

## Abstract

**Background:**

This study aimed to characterize the contemporary landscape of peritoneal dialysis (PD) research published between 2016 and 2025 using PubMed-indexed publications.

**Methods:**

This bibliometric and text-mining study analyzed publications related to PD indexed in PubMed from January 1, 2016, to December 31, 2025. Author keywords were standardized using text normalization. Abstracts were preprocessed using tokenization, stop-word removal, and term normalization to improve topic-modeling performance. Topic modeling was then applied to the processed abstracts to identify latent research themes.

**Results:**

A total of 9,620 PD-related publications were identified between 2016 and 2025, with the annual output increasing from 838 publications in 2016 to 1,128 in 2025. The mean number of author keywords per publication was 3.89±2.99. A keyword analysis identified hemodialysis, peritonitis, mortality, kidney transplantation, and coronavirus disease 2019 as the major research themes. Co-occurrence network analysis revealed three thematic clusters related to dialysis modalities, PD complications, and patient outcomes. Topic modeling identified nine major research topics, with “Catheter complications” and “Mortality and outcomes” remaining the most prevalent throughout the study period. Notably, the prevalence of “Home dialysis” increased from 14.4% to 19.8%, indicating a growing research interest in patient-centered and home-based treatment strategies.

**Conclusion:**

Emerging interest in home dialysis, patient-centered care, and peritoneal fibrosis suggests a shift toward optimizing long-term treatment experience and technique survival.

## Introduction

The number of patients requiring renal replacement therapy continues to increase worldwide, making the selection of an appropriate treatment modality an increasingly important clinical decision [1]. The available renal replacement therapy modalities include kidney transplantation, hemodialysis, and peritoneal dialysis (PD). Although kidney transplantation is considered the preferred treatment option for many patients requiring renal replacement therapy, the limited availability of donor organs results in most patients requiring dialysis.

Despite the well-established advantages of PD, including greater patient autonomy, treatment flexibility, home-based care, and potential economic benefits, its use remains relatively low in many countries. In South Korea, PD accounts for <5% of all patients receiving dialysis, and hemodialysis remains the predominant treatment modality [2]. This imbalance may be influenced by multiple factors, including patient preferences, healthcare system characteristics, socioeconomic factors, physician practice patterns, and institutional resources [3]. Consequently, increasing PD utilization has become an important objective in contemporary nephrology practice.

Understanding the evolution of PD research may provide valuable insights into factors influencing its adoption and clinical implementation. Over the past decade, advances in dialysis technology, infection prevention strategies, patient-centered care, and home dialysis programs have substantially influenced the practice of PD. Recent research has increasingly focused on frailty, sarcopenia, quality of life, peritoneal membrane preservation, and telemedicine. Bibliometric analysis has emerged as a valuable approach for evaluating research trends and identifying emerging themes in scientific disciplines. Furthermore, recent advances in natural language processing and machine learning have enabled large-scale exploration of research topics through analyses of author keywords and publication abstracts.

Previous bibliometric studies of PD have primarily focused on publication productivity, citation impact, and collaborations among countries and institutions [4-6]. Although these studies have provided valuable information on research output and collaboration patterns, they offer limited insights into how research priorities have evolved over time. By integrating conventional bibliometric methods with topic modeling, the present study extends previous investigations by providing a comprehensive characterization of the temporal evolution of research priorities in PD rather than simply describing publication characteristics or citation patterns. Therefore, this study aimed to characterize the contemporary landscape of PD research published between 2016 and 2025 using PubMed-indexed publications. Bibliometric analysis, keyword co-occurrence network analysis, and latent Dirichlet allocation (LDA) topic modeling were used to identify the major research themes and examine their temporal evolution.

## Methods

**Ethics statement:** Ethical approval was not required for this study because it did not involve human participants, human data, or animal subjects.

### 1. Data source and study selection

This bibliometric and text-mining study analyzed publications related to PD indexed in PubMed between January 1, 2016, and December 31, 2025. Literature retrieval was performed using Entrez Programming Utilities provided by the National Center for Biotechnology Information. The literature search and data extraction were conducted on May 28, 2026, and all subsequent analyses were performed using the dataset retrieved on that date. Publications containing the term “peritoneal dialysis” in the title or abstract were identified and retrieved. Bibliographic information, including publication year, title, abstract, and author keywords, was extracted for subsequent analyses. A total of 9,620 publications were identified during the study period. Among these, 8,801 contained abstracts and 7,185 included author-provided keywords. Publications with available abstracts were included in the topic-modeling analyses, whereas those with author-provided keywords were included in the keyword-based bibliometric analyses.

### 2. Data preprocessing

Data preprocessing was performed using Python ver. 3.11 (Python Software Foundation, Wilmington, DE, USA). Author keywords were converted to lowercase keywords and standardized using text normalization procedures. Synonymous terms and spelling variants were consolidated into standardized terms (e.g., “haemodialysis” and “hemodialysis”). General nephrology terms, methodological terms, and other noninformative keywords were excluded using a predefined stop-word list. Abstracts were preprocessed using tokenization, stop-word removal, and term normalization to improve topic-modeling performance.

### 3. Bibliometric analysis

Annual publication counts were calculated to evaluate the publication trends over time. Author keyword analyses included frequency analysis, temporal trajectory heatmaps, and growth rate analysis to identify emerging research topics. The keyword growth rate was calculated as follows: 100×(Count_2025_−Count_2016_)/(Count_2016_+1), where a pseudocount of one was added to the denominator to avoid division by zero for keywords that were absent in 2016. Keyword co-occurrence networks were constructed based on the frequency with which keywords co-occurred in individual publications. Community detection algorithms were applied to identify major thematic clusters within the keyword network.

### 4. Topic-modeling and thematic analysis

LDA topic modeling was applied to the publication abstracts to identify latent research themes. The optimal number of topics was determined by comparing the C_v coherence scores of the LDA models containing two to 12 topics. Each candidate model was trained using 10 passes and 100 iterations with the random seed fixed at 42 to ensure reproducibility. The nine-topic model achieved the highest C_v coherence score and was, therefore, selected for the final analysis. The final LDA model was subsequently retrained using 20 passes and 200 iterations. Each publication was assigned to its dominant topic according to the highest topic probability. Topic labels were assigned based on the most representative keywords within each topic and determined through a consensus between two nephrologists. The annual topic prevalence was calculated to evaluate temporal changes in research priorities between 2016 and 2025. All statistical analyses, network analyses, topic-modeling procedures, and data visualizations were performed using Python libraries including pandas, Matplotlib, NetworkX, Gensim, and related packages.

## Results

### 1. Publication characteristics

A total of 9,620 publications related to PD, published between 2016 and 2025, were identified in PubMed. The mean number of author keywords per publication was 3.89±2.99. Overall, the annual number of PD-related publications increased during the study period. In 2016, 838 publications were identified, followed by 806 in 2017, 797 in 2018, 865 in 2019, and 973 in 2020. Publication output further increased to 1,083 in 2021 and 1,092 in 2022, followed by 986 and 1,052 publications in 2023 and 2024, respectively. The highest annual output of 1,128 publications was recorded in 2025.

### 2. Keyword analysis

The most frequently occurring author keywords were hemodialysis, peritonitis, mortality, kidney transplantation, children, coronavirus disease 2019 (COVID-19), acute kidney injury, continuous ambulatory PD, PD catheter, and peritoneal fibrosis (Fig. 1). Keyword trajectory analysis demonstrated sustained prominence of hemodialysis, peritonitis, and mortality throughout the study period (Fig. 2A). In contrast, several keywords showed a marked increase in frequency over time, including COVID-19, peritoneal fibrosis, automated PD, home hemodialysis, and diabetes mellitus (Fig. 2B).

### 3. Keyword co-occurrence network analysis

A keyword co-occurrence network analysis based on the top 20 keywords identified three major thematic clusters (Fig. 3). Cluster 1 was centered on hemodialysis, kidney transplantation, acute kidney injury, COVID-19, children, quality of life, and home dialysis. Cluster 2 comprised peritonitis, PD catheter, technique failure, outcomes, continuous ambulatory PD, automated PD, and risk factors, representing studies related to infectious complications and PD technique outcomes. Cluster 3 included mortality, cardiovascular disease, diabetes mellitus, inflammation, nutrition, and peritoneal fibrosis, reflecting research on patient outcomes and long-term complications.

### 4. Topic-modeling analysis

Coherence score analysis identified nine topics as the optimal number for LDA modeling (Supplementary Fig. 1). The identified topics were “Mortality and outcomes” (Topic 1), “Catheter complications” (Topic 2), “Nutritional status and sarcopenia” (Topic 3), “Evidence-based treatment” (Topic 4), “Home dialysis” (Topic 5), “Peritoneal membrane function” (Topic 6), “Quality of life and aging” (Topic 7), “Peritonitis and treatment” (Topic 8), and “Peritoneal mesothelial cell and fibrosis” (Topic 9) (Table 1).

The most prevalent topic was “Catheter complications,” accounting for approximately 20% to 25% of publications annually, followed by “Mortality and outcomes” (Fig. 4A). “Home dialysis,” “Quality of life and aging,” and “Peritoneal membrane function” represented topics of intermediate prevalence, whereas “Nutritional status and sarcopenia” accounted for a relatively small proportion of the literature.

The topic-prevalence analysis demonstrated that “Catheter complications” and “Mortality and outcomes” remained the dominant research themes throughout the study period, each accounting for approximately 20% of the publications annually (Fig. 4B). The prevalence of the “Home dialysis” topic increased steadily from 14.4% in 2016 to 19.8% in 2025, indicating a growing research interest in patient-centered and home-based treatment strategies. In contrast, the relative prevalence of “Peritoneal membrane function” declined over time. “Peritonitis and treatment” remained a persistent research priority, whereas “Evidence-based treatment” and “Quality of life and aging” showed a gradual increase in prevalence. Collectively, these findings suggest that contemporary PD research has evolved from a predominant focus on infection- and technique-related complications to a broader interest in home dialysis, patient-centered care, and long-term outcomes.

## Discussion

This study characterized the publication trends and thematic evolution of PD research over a 10-year period using bibliometric and text-mining approaches. Patient outcomes remained a central focus of PD research, as evidenced by the sustained prominence of “Mortality and outcomes”-related topics throughout the study period. Peritonitis also remained a major area of investigation, reflecting its persistent clinical importance as a leading cause of technique failure, hospitalization, and transition to hemodialysis. In contrast, studies focusing on peritoneal membrane function and transport characteristics showed a gradual decline in the relative prevalence over time.

The prominence of hemodialysis among the frequently occurring keywords suggests that a substantial proportion of PD-related studies were conducted in the context of comparisons with hemodialysis or broader kidney replacement therapy populations, including kidney transplantation. As hemodialysis remains the most widely used dialysis modality worldwide, numerous studies have compared PD with hemodialysis in terms of survival, quality of life, cardiovascular outcomes, and healthcare utilization. These findings underscore the continued relevance of evaluating PD within the broader landscape of kidney replacement therapy and reflect the ongoing efforts to optimize modality selection for patients with end-stage kidney disease. The sustained prominence of peritonitis- and mortality-related keywords indicates that infectious complications and patient survival continue to be the major research priorities in PD. Despite substantial advances in connection systems, catheter management, and preventive strategies, peritonitis remains a leading cause of technique failure and the transition to hemodialysis [7]. Similarly, mortality continues to be a key clinical outcome in PD research, reflecting an ongoing emphasis on improving long-term patient outcomes. Collectively, these findings indicate that infection prevention and survival remain fundamental priorities, despite evolving clinical practice. Another notable finding was the emergence of COVID-19–related publications alongside the increasing prominence of peritoneal fibrosis. The temporary increase in COVID-19–related research most likely reflects the advantages of home-based dialysis during the pandemic as well as the need to evaluate the impact of severe acute respiratory syndrome coronavirus 2 infection on patients receiving PD [8].

In contrast, growing interest in peritoneal fibrosis appears to represent a sustained shift in research priorities. As improvements in patient survival and technique longevity have extended the duration of PD treatment, greater attention has been directed toward the long-term structural and functional changes in the peritoneal membrane. This trend reflects the increasing recognition that the preservation of peritoneal membrane integrity is essential for long-term technique survival and optimal patient outcomes. A discrepancy was observed between keyword trends and topic prevalence. Peritoneal fibrosis was among the fastest-growing keywords, indicating increasing research attention on peritoneal membrane preservation and the mechanisms of long-term membrane injury. However, fibrosis-related topics account for a relatively small proportion of the literature on LDA-based topic analyses. This observation suggests that, although peritoneal fibrosis has become an increasingly active area of investigation, it remains a specialized research area rather than a dominant theme within the broader PD literature. In contrast, topics related to mortality, peritonitis, catheter complications, and home dialysis continued to account for a substantially larger proportion of publications.

At the same time, the relative prevalence of studies focusing on peritoneal membrane function declined over the study period. Although the preservation of peritoneal membrane integrity remains a fundamental determinant of long-term PD success, this trend may reflect a broader shift in research priorities rather than the diminished clinical importance of membrane biology. In recent years, the International Society for Peritoneal Dialysis has increasingly emphasized patient-centered care, highlighting quality of life, symptom burden, shared decision-making, and individualized treatment goals alongside traditional clinical outcomes [9,10]. Consistent with this shift, contemporary PD research has expanded beyond physiological and membrane-related parameters to encompass broader aspects of patient well-being and the treatment experience. This interpretation is further supported by the increasing prominence of topics related to home dialysis, quality of life, and patient-centered care, accompanied by the declining relative prevalence of studies on peritoneal membrane function. These findings do not imply a reduced interest in preserving the peritoneal membrane. Rather, they suggest the emergence of two complementary research directions: continued investigation of the biological mechanisms underlying membrane injury, as reflected by the growing interest in peritoneal fibrosis, and an expanding emphasis on patient-centered outcomes and individualized care. Future studies should integrate mechanistic investigations of membrane preservation and fibrosis with patient-reported outcomes and quality-of-life measures to optimize long-term PD care and technique survival.

Compared with previous bibliometric studies on PD, the present study offers several methodological and conceptual advances. Earlier investigations primarily focused on publication productivity, citation characteristics, highly cited articles, and research collaborations, thereby providing valuable descriptive insights into the development of this field [4-6]. In contrast, the present study integrated conventional bibliometric analyses with LDA topic modeling to characterize not only publication trends, but also the temporal evolution of underlying research themes. This approach has enabled the identification of persistent research priorities (such as mortality and peritonitis) and emerging areas of interest, including home dialysis, patient-centered care, and peritoneal fibrosis. Consequently, these findings complement previous bibliometric studies by providing a more comprehensive understanding of how PD research priorities have evolved over the past decade.

The primary objective of this study was to characterize the contemporary PD research landscape using text mining and topic modeling. Accordingly, a broad search strategy based on the occurrence of the term “peritoneal dialysis” in the title or abstract was adopted rather than restricting retrieval to Medical Subject Headings (MeSH) indexing. Although MeSH-based searches provide excellent specificity, they may fail to capture recently published articles, publications that have not yet been assigned MeSH terms, and studies in which PD represents a major research focus but is not designated as the primary MeSH heading. Because topic modeling aims to identify latent semantic structures within a literature corpus, maximizing corpus inclusiveness is preferable. Furthermore, the proportion of records lacking MeSH terms increased over the study period from 14.4% in 2016 to 39.5% in 2025 (14.4% in 2016, 14.8% in 2017, 15.8% in 2018, 19.2% in 2019, 20.4% in 2020, 28.3% in 2021, 27.0% in 2022, 26.1% in 2023, 32.4% in 2024, and 39.5% in 2025). Given the substantial proportion of publications without MeSH indexing, particularly in recent years, restricting the search to MeSH terms alone would likely have excluded a considerable number of relevant studies. Therefore, the inclusion of title- and abstract-based searches together with author-provided keywords improved the comprehensiveness of the dataset.

The exceptionally high growth rate observed for COVID-19 should be interpreted with caution as it primarily reflects the abrupt emergence of the pandemic rather than a sustained shift in research priorities. Because COVID-19–related publications were virtually absent before 2020, percentage-based growth estimates inevitably exaggerated this apparent increase. Accordingly, keyword growth rates should be interpreted along with an annual keyword trajectory heat map, which provides a more informative assessment of temporal research trends. Collectively, these findings indicate that COVID-19–related publications represent a transient, event-driven surge in research activity, rather than a sustained long-term research direction.

This study had some limitations. First, only PubMed-indexed publications were analyzed. Although PubMed provides comprehensive coverage of biomedical literature and standardized indexing suitable for bibliometric and text-mining analyses, relevant studies indexed exclusively in databases such as Scopus, Web of Science, or Embase may not have been captured. Second, the search strategy relied on the term “peritoneal dialysis” appearing in the title or abstract, which may have excluded studies highly relevant to PD that did not explicitly use this terminology. Third, keyword analyses were based on author-provided keywords inherently influenced by individual preferences and journal-specific practices. Although extensive text normalization and synonym mapping were performed, some degree of heterogeneity may have persisted. Fourth, the results of topic modeling are inherently influenced by text preprocessing, the selection of topic number, and model assumptions. Although the optimal number of topics was determined using coherence score analysis, and topic labels were assigned by consensus between two nephrologists, some degree of subjectivity in topic interpretation was unavoidable. Finally, bibliometric analyses can characterize research trends and thematic evolution, but cannot directly assess the methodological quality, scientific impact, or clinical significance of individual studies. Accordingly, these findings should be interpreted as reflecting patterns of research activity rather than as evidence of clinical importance.

In conclusion, PD research has evolved substantially over the past decade while maintaining a strong focus on patient outcomes and peritonitis. The emerging interest in home dialysis, patient-centered care, and peritoneal fibrosis suggests a shift toward optimizing long-term treatment experience and technique survival. These findings provide a comprehensive overview of evolving research priorities in PD and may help inform future research.

## Figures and Tables

**Fig. 1.**
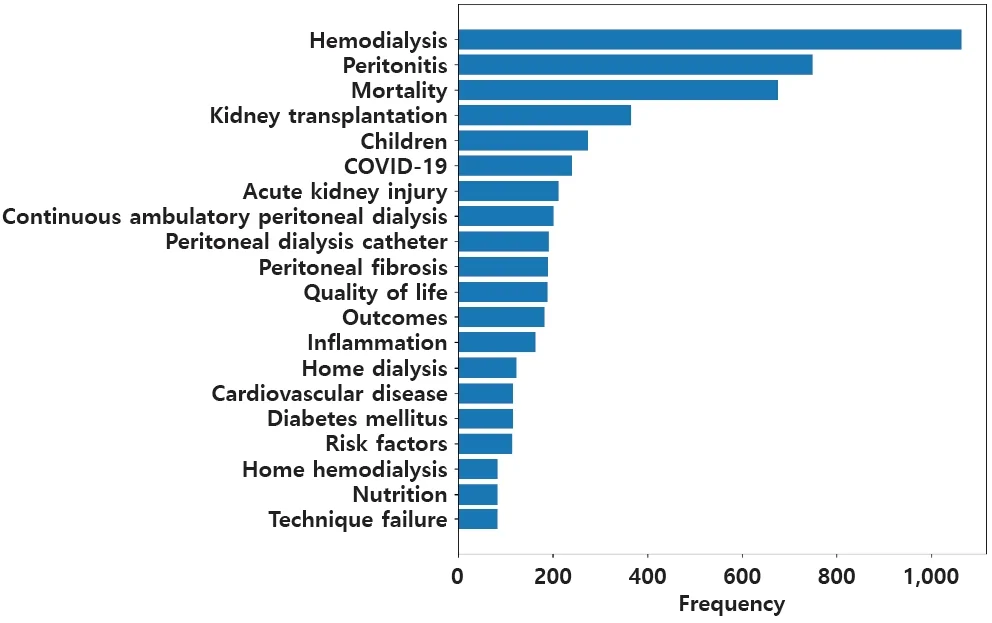
Top 20 author keywords from 2016 to 2025. COVID-19, coronavirus disease 2019.

**Fig. 2.**
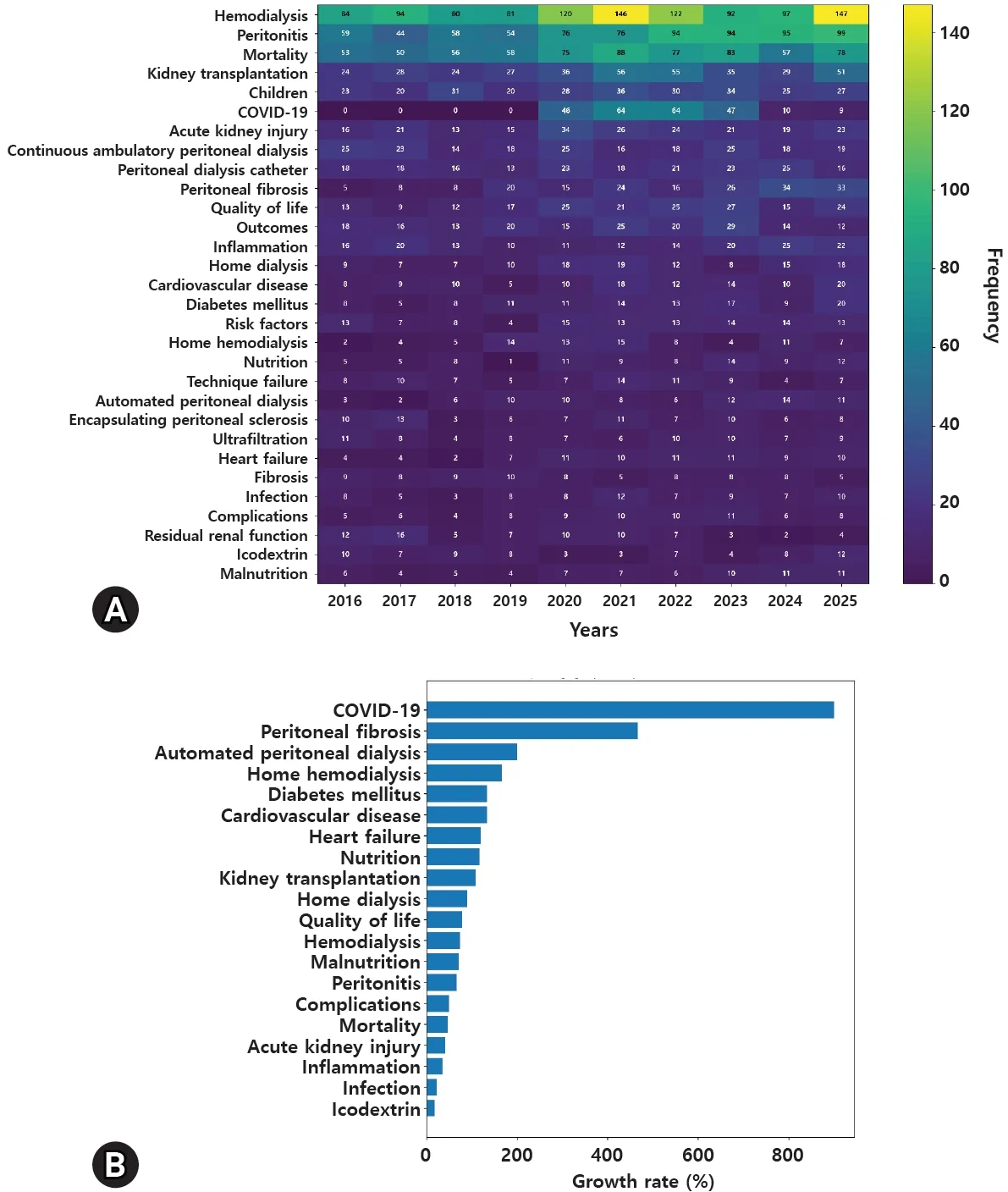
(A) Keyword trajectory heatmap and (B) top emerging keywords ranked by growth rate, 2016 to 2025. COVID-19, coronavirus disease 2019.

**Fig. 3.**
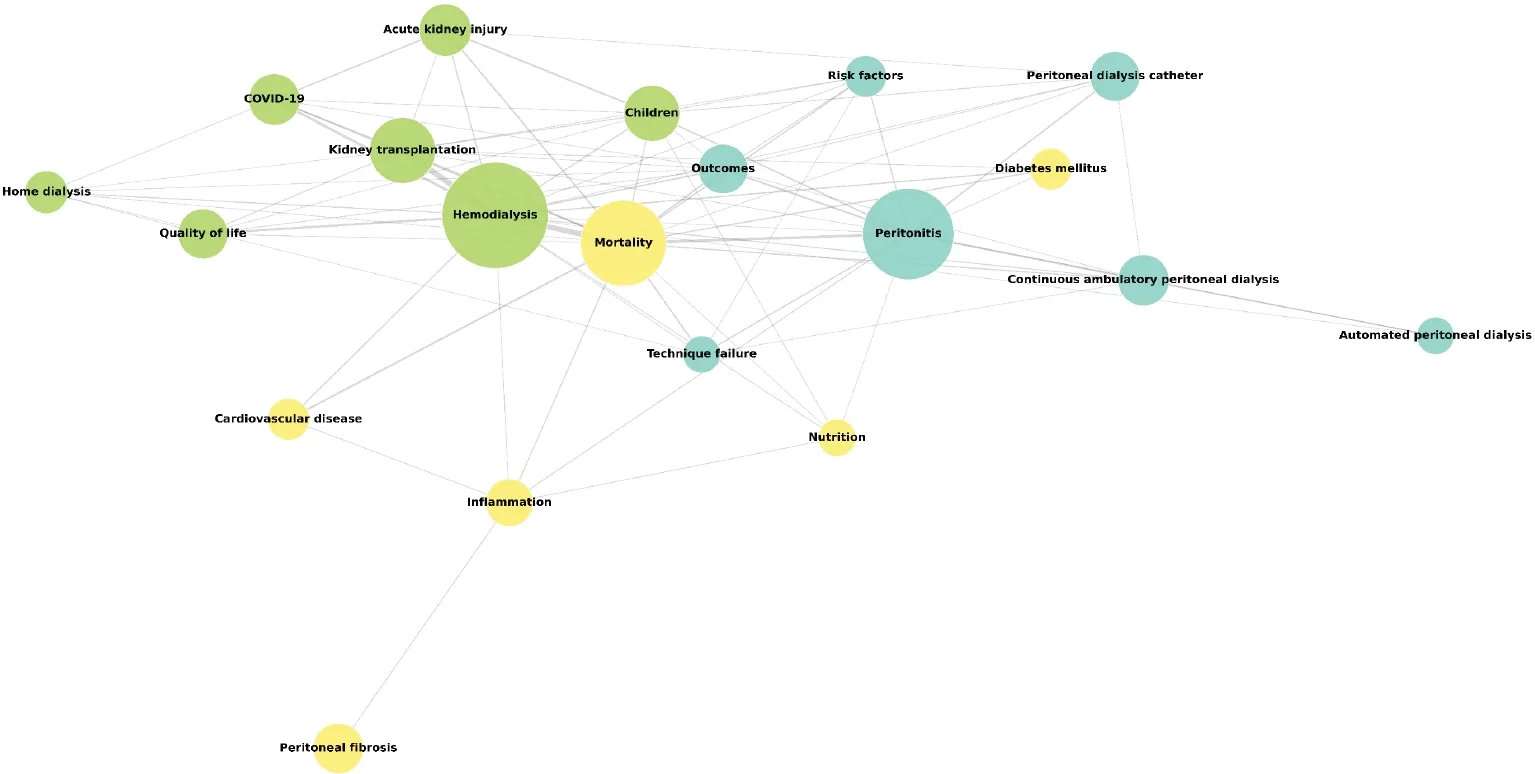
Keyword co-occurrence network, 2016 to 2025. Keywords with the same color belong to the same cluster, representing groups of terms with relatively strong co-occurrence relationships.

**Fig. 4.**
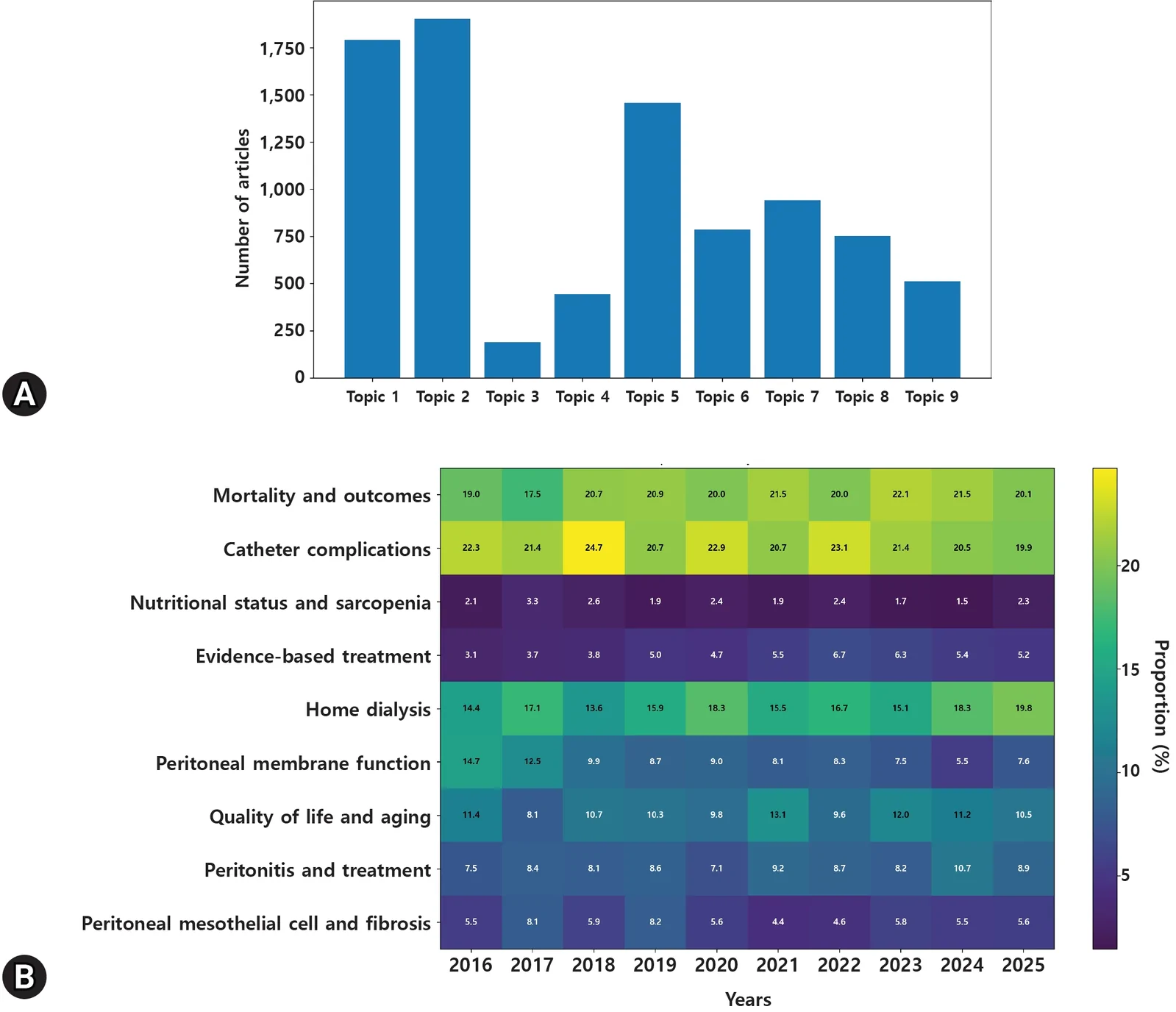
(A) Number of publications by dominant topic and (B) temporal trends in topic prevalence in peritoneal dialysis research.

**Table 1.** Latent Dirichlet allocation-derived research topics in peritoneal dialysis literature (2016–2025)

Topic	Topic label	Representative keyword
Topic 1	Mortality and outcomes	Mortality, age, outcomes, factors, cardiovascular, regression, serum, cohort, ratio, all-cause
Topic 2	Catheter complications	Catheter, complications, disease, therapy, case, acute, failure, days, hemodialysis, AKI
Topic 3	Nutritional status and sarcopenia	Body, mass, nutritional, muscle, sarcopenia, status, intake, volume, dietary, protein
Topic 4	Evidence-based treatment	Sup, sub, review, treatment, evidence, outcomes, trials, dose, effects, randomized
Topic 5	Home dialysis	Care, home, health, hemodialysis, disease, treatment, outcomes, therapy, management, access
Topic 6	Peritoneal membrane function	Glucose, serum, function, dialysate, plasma, ultrafiltration, concentration, blood, transport, creatinine
Topic 7	Quality of life and aging	CKD, disease, hemodialysis, chronic, quality, prevalence, life, age, significant, factors
Topic 8	Peritonitis and treatment	Peritonitis, infection, treatment, infections, episodes, CAPD, catheter, case, cases, PDAP
Topic 9	Peritoneal mesothelial cell and fibrosis	Fibrosis, cells, expression, mesothelial, cell, EPS, TGF, treatment, induced, factor

AKI, acute kidney injury; CKD, chronic kidney disease; CAPD, continuous ambulatory peritoneal dialysis; PDAP, peritoneal dialysis-associated peritonitis; EPS, encapsulating peritoneal sclerosis; TGF, transforming growth factor.

## Supplementary materials

Supplementary Fig. 1 can be found at https://doi.org/10.12701/jyms.2026.43.58.

Supplementary Fig. 1. Coherence score for determination of the optimal number of latent Dirichlet allocation topics.
