# Supplementary material for: Research trends in peritoneal dialysis literature: a bibliometric and topic-modeling analysis of PubMed publications from 2016 to 2025
Source: J Yeungnam Med Sci. 2026 Aug 21;43:58. doi: 10.12701/jyms.2026.43.58 (PMC13681082; doi:10.12701/jyms.2026.43.58)
Supplement: Supplementary file 1 [file jyms-2026-43-58-Supplementary-Fig-1.pdf]

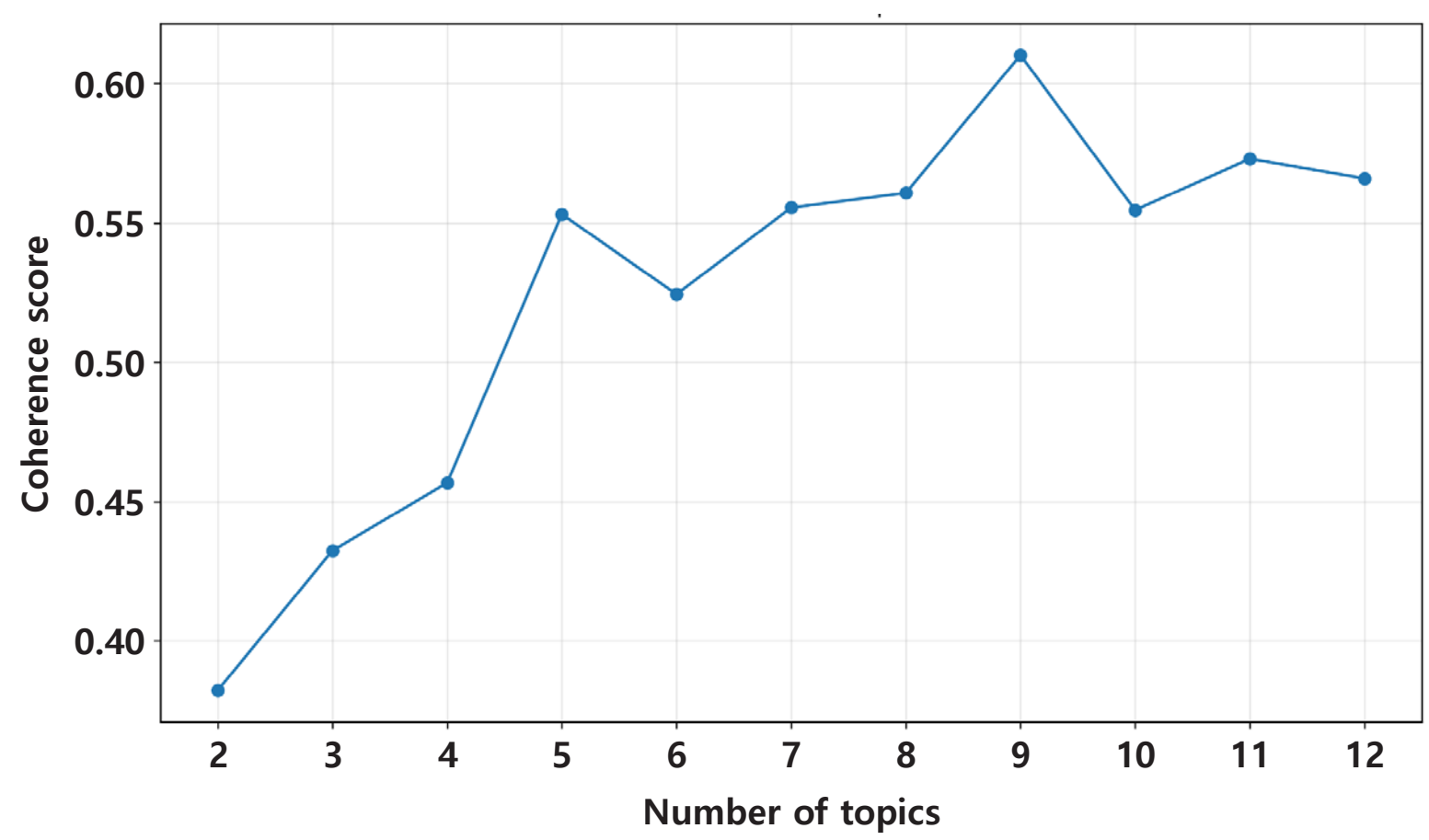

Supplementary Fig. 1. Coherence score for determination of the optimal number of latent Dirichlet allocation topics.

https://doi.org/10.12701/jyms.2026.43.58
